# Efficacy of Mucosamin Spray as an Adjunct for Prevention of Oral Mucositis in Children under Chemotherapy: A Two-Center Randomized Clinical Trial

**DOI:** 10.30476/dentjods.2023.98910.2116

**Published:** 2024-09-01

**Authors:** Fatemeh Ghoroubi, Mandana Alamdari Mahd, Azim Mehrvar, Bibi Shahin Shamsian, Sara Tavassoli-Hojjati

**Affiliations:** 1 Dentist, School of Dentistry, Islamic Azad University of Tehran, Tehran, Iran; 2 Postgraduate Student, Dept. of Pediatrics, School of Dentistry, Islamic Azad University of Tehran, Tehran, Iran; 3 Mahak Hematology Oncology Research Center (Mahak-HORC), Mahak Hospital, Shahid Beheshti University of Medical Sciences, Tehran, Iran; 4 Pediatric Congenital Disorders Research Center, Mofid Children Hospital, Shahid Beheshti University of Medical Sciences, Tehran, Iran; 5 Dept. of Pediatric Dentistry, Faculty of Dentistry, Tehran Medical Sciences, Islamic Azad University, Tehran, Iran

**Keywords:** Oral Mucositis, Leukemia, Lymphoma, Chemotherapy, Mucosamin

## Abstract

**Statement of the Problem:**

Chemotherapy-induced mucositis is the most common complication during cancer treatment. This complication can lead to pain, increased risk of infection and malnutrition. Therefore, it is important to find a solution to reduce the severity and duration of side effects.

**Purpose:**

This study aimed to assess the efficacy of Mucosamin spray as an adjunct for prevention of oral mucositis in children under chemotherapy.

**Materials and Method:**

This parallel-design clinical trial evaluated 48 patients aged 5 to 15 years with leukemia and lymphoma presenting to the Hematology Department of Mofid and Mahak Hospitals. The patients were randomly divided into two groups (n=24). Before starting chemotherapy, all patients received oral hygiene instructions (toothbrushing without flossing). Patients in both groups were requested to use a mouthwash composed of nystatin, aluminum-magnesium hydroxide suspension (aluminum MgS), and diphenhydramine for 1 min every morning on a daily basis starting the day before treatment. Patients in the intervention group were also requested to spray their entire oral mucosa with Mucosamin spray 3 times a day in addition to using the mouthwash. Patients were requested to refrain from eating and drinking for 1h after spraying. The patients were clinically examined by a senior dental student once every other day for 20 days regarding the occurrence, severity, and duration of oral mucositis. Data were analyzed using the Chi-square and Mann-Whitney tests, Kaplan-Meier survival analysis, and log rank test.

**Results:**

No significant difference was noted between the two groups in the incidence, severity, or time of development of mucositis (*p*> 0.05). The 7-day non-recovery percentage was 72.7% (SE= 13.4) and 25.0% (SE= 15.3) in the control and test groups, respectively, indicating shorter duration (faster healing) of mucositis in the intervention group (*p*= 0.01).

**Conclusion:**

Within the limitations of this study, it seems that prophylactic application of Mucosamin spray can shorten the course of oral mucositis, in case of its occurrence.

## Introduction

Chemotherapy-induced mucositis is the most common complication during cancer treatment [ 1 ]. Oral mucositis occurs following the production of reactive oxygen species in the submucosa [ 2 ]. It often manifests 5 to 10 days after the initiation of chemotherapy, and it is characterized by erythema, oral mucosal sores, and inflammation, which lead to painful, ulcerative, and hemorrhagic ulcers [ 3 ]. The complications of oral mucositis include pain, increased risk of local and systemic infections, bleeding, and malnutrition. These complications may interfere with the course of treatment and aggravate the prognosis [ 4 - 5 ]. The efficacy of mouthwashes, dietary supplements, anti-oxidants, and hyaluronic acid products for prevention and treatment of chemotherapy-induced mucositis has been extensively studied. Nonetheless, no consensus has been reached on a definite treatment or guideline for management of oral mucositis.

Mucosamin spray (Professional Dietetics Co, Italy) is a recent treatment strategy for oral mucositis. Its manufacturer emphasizes on its preventive and therapeutic effects on chemotherapy- and radiotherapy-induced mucositis [ 2 ]. It has been claimed that there was no reports of side effects or interactions with drugs or medicinal substances. Mucosamin spray contains hyaluronic acid and natural amino acids. Attachment of hyaluronic acid with high molecular weight to free radical catalyzers causes the breakdown of hyaluronic acid into smaller pieces, and subsequent inactivation of reactive oxygen species [ 6 ]. Moreover, hyaluronic acid has a protective effect on oral mucosa, enhances wound healing (by inducing the migration of fibroblasts), decreases pain and wound size, and accelerates the healing process [ 7 ]. In addition, evidence shows that the Mucosamin spray decreases the incidence of mucositis, enhances healing, and alleviates pain [ 8 ]. Moreover, it has been demonstrated that use of a hyaluronic acid-based mouthwash in addition to Mucosamin spray decreases the severity of mucositis [ 9 - 10 ]. Another study reported that Mucosamin spray decreased the severity of mucositis and accelerated healing in patients with stem cell transplantation [ 11 ]. In addition to the therapeutic effects of Mucosamin, its preventive effects on development of mucositis were confirmed in a study on a small number of adults [ 2 ]. Nonetheless, the results of the available literature on the efficacy of hyaluronic acid for prevention and treatment of mucositis are controversial, and a general consensus has not yet been reached regarding its optimal efficacy [ 12 ]. Thus, this clinical trial aimed to assess the efficacy of Mucosamin spray for prevention of chemotherapy-induced oral mucositis in 5-15-year-old patients with lymphoma and leukemia hospitalized in two specialty hospitals in Tehran.

## Materials and Method

This parallel-design randomized clinical trial was conducted in two hospital settings. The study was approved by the Ethics Committee of School of Dentistry, Islamic Azad University of Medical Sciences (IR.IAU. DENTAL.REC.1398.017) and registered in the Iranian Registry of Clinical Trials (IRCT20190705044106N1). Primary evaluation of medical records of 50 patients admitted to Mofid Children’s Hospital from 2019 to 2020 revealed the incidence of mucositis to be 60% in patients (Table 1). If the intended intervention can decrease this rate by one-third, testing this hypothesis would require 20 samples in each group, assuming type one error of 5% and study power of 80%. Considering 20% dropouts, 24 patients were recruited for each group (a total of 48 patients). Thus, 48 patients were selected among those between 5-15 years with leukemia and lymphoma presenting to the Hematology Department of Mahak and Mofid Hospitals. The patients were selected by the supervisor of the department and were randomly divided into two groups of test and control (n=24). Patients admitted with an odd number were assigned to the test group and those admitted with an even number were assigned to the control group (the last two patients were allocated to the control group because the 24 members of the test group had been already recruited). Patients under orthodontic treatment were excluded, and those with defective dental restorations were either excluded or their restorations were replaced prior to the initiation of treatment. Patients with autoimmune diseases were also excluded (due to impaired wound healing). All Patients received the medication according to the treatment protocol during the induction phase. The patients with acute lymphocytic leukemia (ALL) received dexamethasone or oral prednisone, vincristine, adriamycin, lasparaginase followed by central nervous system (CAN) intrathecal prophylaxis and patients with lymphoma received dexamethasone or oral prednisone, vincristine, cyclophosphamide followed by CNS intrathecal prophylaxis and patients with acute myeloid leukemia (AML) received the DAT protocol, which includes cytoxan (Ara C), daunorubicin, thioguanine followed by CNS intrathecal, or the MRC protocol, which includes daunorubicin, cytoxan, VP16 (Etoposide) followed by CNS intrathecal prophylaxis [ 13 ]. Written informed consent for participation in the study was obtained from the parents and patients were free to leave the study at any time they wished. In addition, for the purpose of standardization, all patients received oral hygiene instructions (correct toothbrushing without flossing) prior to the initiation of chemotherapy. Moreover, all patients were instructed to have a non-acidic diet and refrained from eating spicy foods during the course of chemotherapy.

**Table 1:** Frequency of mucositis in patients with leukemia and lymphoma in patients admitted to Mofid Children’s Hospital from 2019 to 2020

Incidence	Presence	Absence	Total (Percentage)
Condition			
ALL	15	6	21
AML	2	5	7
Hodgkin's lymphoma	6	7	13
Non-Hodgkin's lymphoma	7	2	9
Total	30(60%)	20(40%)	50 (100%)

ALL: acute lymphoblastic leukemia, AML: acute myeloid leukemia

All patients in the test and control groups received a mouthwash composed of 10 drops of nystatin, aluminum magnesium hydroxide suspension (aluminum MgS), and diphenhydramine in 1:1:1 ratio, and were instructed to swish 10mL of the mouthwash every morning after breakfast for 1 min and refrain from eating and drinking for 1 h after it, starting one day before the initiation of treatment (induction phase) to prevent mucositis. Mucosamin (Mucosamin Spray, Professional Dietetics Co, Italy) was sprayed into the patients' mouths in the test group from one day before the start of chemotherapy until 20 days later by the hospital nurse for 3 times a day, in addition to using the mouthwash. According to the manufacturer’s instructions, the patients were requested to remove any foreign body from the mouth by rinsing the mouth prior to spraying. Moreover, a uniform layer of gel had to be directly sprayed by the applicator on the entire oral mucosa. Mucosamin had to remain in the oral cavity for a minimum of 2 min, and the patients were refrained from eating and drinking for 1h after using it. The timing of using the mouthwash and spray by the test group was as follows.

**Figure 1:**
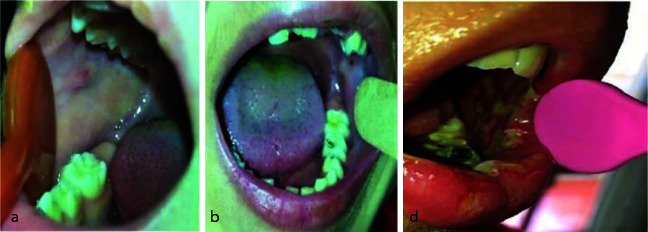
Severity of mucositis in patients, **a:** Grade 2 mucositis in the test group, **b:** Grade 2 mucositis in the control group, **c:** Grade 3 mucositis in the control group

After breakfast, the mouthwash had to be swished at 7:30 a.m., and then the Mucosamin spray had to be used at 8:30 a.m. Also, the Mucosamin spray had to be used after lunch and after dinner. A senior dental student, trained for diagnosis of mucositis, who was blinded to the group allocation of patients, examined the oral cavity of patients every other day for a minimum period of 20 days. The evaluation started on 24th of August 2021 and lasted for seven months. The incidence and severity of mucositis were determined and recorded according to the classification by the World Health Organization (WHO) (Table 2, Figure 1) [ 14 ]. Presence of erythema and oral ulcers was clinically evaluated. The primary outcomes of the study were to evaluate the difference of mucositis incidence and severity between two groups and the effect of Mucosamin spray on decreasing the duration of oral mucositis, delaying the onset of oral mucositis between two groups were the secondary outcomes.

**Table 2:** Grade of mucositis according to the World Health Organization (WHO)

Grade	Clinical symptoms
0	Absence of signs and symptoms of mucositis
1	Erythema, no ulceration
2	Erythema and ulceration, being able to swallow solid food
3	Erythema and ulceration, being able to swallow liquids, not being able to swallow solid food
4	Erythema and ulceration, not being able to swallow liquids or solid food

Data were transferred to SPSS version 24 and analyzed using the Chi-square test, Mann-Whitney test, Kaplan-Meier curve (survival analysis), and log rank test at *p*< 0.05 level of significance.

## Results

### Participant flow

**Figure 2:**
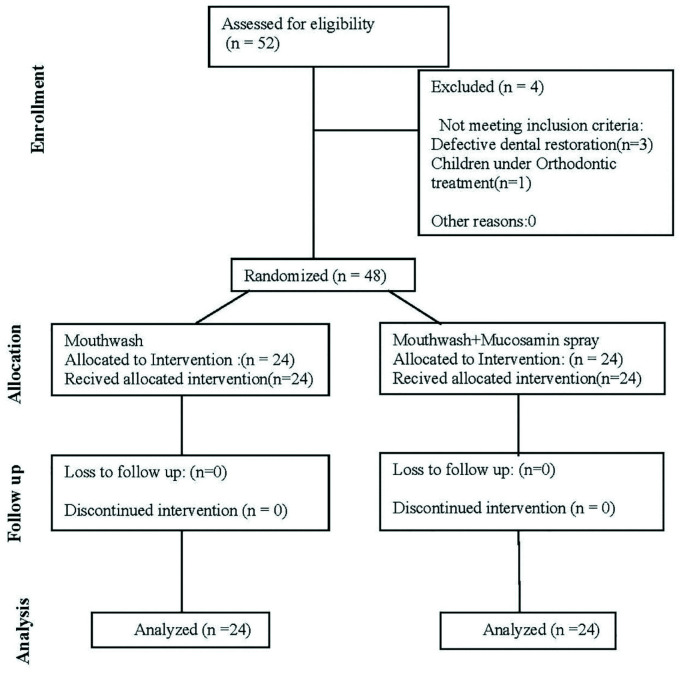
CONSORT flow diagram of the study

The sample consisted of 5-15 years old children with leukemia. Figure 2 shows the CONSORT flow diagram of patient selection and allocation to the study groups. 

In this clinical trial, the average age in the control and intervention group was respectively 8.03±3.32 and 7.03±2.68. In both groups, 19 males and 5 females participated, and overall, there were 7 patients with AML, 34 patients with ALL, and 7 patients with lymphoma.

The incidence of mucositis was 33.3% in the test and 50.0% in the control group; this difference was not statistically significant (*p*= 0.24). Patients who received the Mucosamin spray had 50% lower risk of development of mucositis; however, this finding could not be generalized (OR=0.50; 95% CI:0.16-1.61). Patients in the test group were at higher risk of developing grades I and II mucositis; but this difference was not statistically significant (*p*= 0.24, Table 3).

**Table 3:** Frequency distribution of severity of mucositis in the test and control groups

Grade of mucositis		0 (absent)	1	2	3	Total
Study groups	Control	12 (50.0%)	5 (20.8%)	6 (25.0%)	1(4.2%)	24 (100.0%)
	Test	16(66.7%)	4 (16.7%)	3 (12.5%)	1 (4.2%)	24 (100.0%)
Total		28 (58.3%)	9 (18.8%)	9 (18.8%)	2 (4.2%)	48 (100.0%)

Data regarding the time of onset of mucositis and its duration in patients were not normally distributed. Thus, they were reported as the median and interquartile range (IQR). Assessment of patients with mucositis in the two groups revealed that mucositis occurred in half of the patients in the test group in less than 14 days while it occurred in less than 10 days in half of the patients in the control group; however, the difference in the time of onset of mucositis was not significant between the two groups (*p*= 0.11).

Assessment of duration of mucositis (in patients who developed mucositis) revealed that it lasted for less than 4 days in half of the patients in the test group (IQR =2.00-8.00, median: 4.00) while it lasted for 9.5 days in the control group; this difference was not significant (*p*= 0.07, Table 4). 

**Table 4:** Time of onset and duration of mucositis in patients who developed mucositis in the test and control groups

		Control group (n=12)	Test group (n=8)	*p* value
Time of onset of mucositis	Median	10	14	0.11
	Interquartile range	8-11	10-15.5	
Duration of mucositis	Median	9.5	4	0.07
	Interquartile range	6-13	2-8	

The results of survival analysis by the Kaplan-Meier curve revealed that the disease-free percentage in the first 10 days after the onset of chemotherapy was 87.5% (SE=6.8) in the test and 79.2% (SE=8.3) in the control group. In other words, at the 10-day follow-up, around 12% of the test group and 21% of the control group developed mucositis while others did not develop mucositis in the first 10 days. These results indicated earlier occurrence of mucositis in the control group; however, this difference did not reach statistical significance (*p*= 0.17). 

In addition, the Kaplan-Meier curve was used to analyze the difference in the duration of mucositis (its recovery) in the two groups. The results showed that the percentage of non-recovery at 7 days was 72.7% (SE: 13.4) in the control and 25.0% (SE:15.3) in the test group. This difference indicated faster recovery of mucositis in the test group (*p*= 0.01).

## Discussion

Almost all patients under chemotherapy and radiotherapy develop oral ulcerative mucositis [ 15 ]. Nonetheless, no definite treatment or effective guideline has been offered for prevention or management of oral mucositis. Considering the high risk of development of mucositis in patients under chemotherapy and radiotherapy, each hospital has its own protocol for prevention and treatment of mucositis. In Mofid Children’s Hospital, this protocol is composed of a mouthwash containing nystatin (antifungal agent), aluminum MgS (anti-acid), and diphenhydramine (anti-emetic) in 1:1:1 ratio, which should be used in an amount of 10 mL. Primary assessment of 50 medical records of patients hospitalized in the Hematology Department of this hospital revealed the incidence of mucositis to be 60%. This finding indicates that despite the continuous use of the mouthwash by patients, most of them still develop and suffer from mucositis. Considering the fact that the protocol of the hospital could not be changed, we decided to use the Mucosamin spray as an adjunct to the mouthwash in our patients. Mucosamin spray contains hyaluronic acid and natural amino acids.

The current results showed that the Mucosamin spray decreased the incidence of mucositis in the test group by 17%; however, this reduction was not statistically significant (*p*= 0.24). Several studies have assessed the efficacy of Mucosamin spray for treatment of oral mucositis, and reported some information about the severity of mucositis, its duration, and severity of pain [ 8 , 11 , 16 ]. Information regarding the preventive effect of Mucosamin spray on the incidence of mucositis is limited. Cirillo *et al*. [ 2 ] in 2015 evaluated the efficacy of Mucosamin spray for prevention of oral mucositis *in vitro* and *in vivo*. After prophylactic treatment with Mucosamin spray in 5 patients under radiotherapy, chemotherapy or both, the results revealed that grade 1 mucositis (according to the WHO classification) only occurred in 1 out of 5 cases. Their results confirmed the optimal efficacy of Mucosamin spray for prevention of oral mucositis. However, it should be noted that their sample size was small, and they did not have a control group. In addition, they evaluated patients over 50 years of age, who were under chemotherapy, radiotherapy, or both. However, our study was conducted on 5-15-year-olds with either leukemia or lymphoma. The methodology of the two studies was also different. In their study, the patients used the spray 4 days prior to the onset of treatment while in the current study the patients started using the spray one day before the onset of chemotherapy. These differences may explain the difference in the results of the two studies. Prophylactic use of Mucosamin spray breaks down the reactive oxygen species and prevents oxidative stress and senescence of fibroblasts. On the other hand, fibroblasts have the responsibility to control the function and survival of keratinocytes. Presence of aged fibroblasts leads to impaired function of keratinocytes. Thus, it may be concluded that prophylactic use of this spray can prevent the initiation of this vicious cycle [ 2 ].

The results regarding the time of onset of mucositis revealed that half of the patients in the test group developed oral mucositis in less than 14 days, while half of the patients in the control group developed oral mucositis in less than 10 days. This difference was not statistically significant (*p*= 0.11). 

With regard to duration of mucositis in patients who developed mucositis, the results showed that half of the patients in the test and control groups had mucositis for 4 and 9.5 days, respectively. However, this difference was not significant (*P*=0.07). Since, statistical analysis was performed on 20 patients who developed mucositis, there is a possibility that this number is not enough to compare the two groups regarding the time of onset or duration of mucositis. Ruggiero *et al*. [ 11 ] evaluated 137 patients who had undergone bone marrow transplantation, and reported faster recovery in patients who used the Mucosamin spray. According to Trotti *et al*. [ 15 ], the risk of oral mucositis is 80% in patients with head and neck cancer who underwent radiotherapy, 40% in those who took chemotherapy, and 100% in patients who had bone marrow transplantation. In this study, our patients underwent chemotherapy for leukemia or lymphoma; however, in the study by Ruggiero *et al*. [ 11 ], all patients had undergone bone marrow transplantation and therefore, had higher risk of development of oral mucositis; thus, the two studies cannot be accurately compared. 

This study also showed that the disease-free percentage in the first 10 days in the test group was higher than that in the control group. This indicates earlier development of mucositis in the control group; however, this difference was not statistically significant (*p*= 0.17). Also, the percentage of 7-day non-recovery was 72.7% (SE=13.4) in the control and 25.0% (SE:15.3) in the test group. This difference indicated faster recovery and shorter duration of mucositis in the test group; this difference was statistically significant (*p*= 0.01). The results of Colella *et al*. [ 8 ] support our findings. They used Mucosamin spray for 27 adult patients under head and neck radiotherapy, and reported faster recovery of mucositis due to the effect of Mucosamin spray. Bardellini *et al*, [ 9 ] in 2016 evaluated 56 patients between 5-18 years with ALL and reported a reduction in severity of mucositis between days 3 and 8 after treatment in the group that used a mouthwash with hyaluronic acid base, compared with the placebo group. As mentioned earlier, Ruggiero *et al*. [ 11 ], evaluated 137 patients under bone marrow transplantation and reported similar results. Patients who used the Mucosamin spray for therapeutic purposes recovered earlier than the other groups. Nasrollahi *et al*. [ 17 ] in 2021 evaluated the effect of Mucosamin in preventing mucositis in 80 patients aged 18-75 years undergoing radiotherapy for oral squamous cell carcinoma. The control group received mouthwash with magnesium, aluminum hydroxide, diphenhydramine, and nystatin, while the intervention group received Mucosamin spray. Weekly mucositis evaluation was performed based on the radiation therapy oncology group (RTOG) scoring criteria. Contrary to the present study, Mucosamin spray significantly reduced the severity of mucositis compared to the control group. Differences in methodology and lack of randomization and blinding were among the reasons for the discrepancy of the results with the present study. Shahrabi *et al*. [ 18 ] in 2022 investigated the effect of Mucosamin spray in preventing mucositis in 60 patients aged 4-18 years undergoing hematopoietic stem cell transplantation. The control group received placebo spray with similar ingredients to Mucosamin, except for hyaluronic acid and amino acids. The follow-up for assessment of mucositis was performed every three days for up to 21 days, and the national cancer institute common terminology criteria for adverse events index (NCI-CTCAE) was used to assess the grade of mucositis. In contrast to the present study, the results showed that Mucosamin spray significantly reduced the prevalence and severity of mucositis compared to the placebo spray and was consistent with our study, demonstrating that the use of Mucosamin spray significantly reduced the duration of mucositis. Differences in the severity grading indices of mucositis and differences in chemotherapy drugs and oral hygiene standardization, which might affect the severity and occurrence of mucositis, are reasons for the different results between the two studies.

To better assess the therapeutic effects of Mucosamin spray, the efficacy of hyaluronic acid and amino acids in its composition should be separately evaluated. Hyaluronic acid is effective in cellular proliferation and differentiation. The amino acids present in the spray increase and reinforce the cellular metabolism by induction of collagen formation and synthesis of extracellular matrix, and accelerate wound healing as such [ 19 ].

This study was the first clinical trial on the preventive effects of Mucosamin spray on development of mucositis in children and adolescents. The patients were selected from two hospitals with strict inclusion and exclusion criteria, and high accuracy. Oral hygiene instruction and medication intake were scheduled according to a timetable. One of the limitations of this study was the lack of placebo spray and it was not possible to blind the patients. It is suggested that future studies may be required on a higher number of patients with different cancer types undergoing chemotherapy and radiotherapy.

## Conclusion

Within the limitations of this study, it seems that prophylactic use of Mucosamin spray can shorten the course of mucositis and enhance its recovery, in case of its occurrence.
